# Alignment philosophy influences trochlea recreation in total knee arthroplasty: a comparative study using image-based robotic technology

**DOI:** 10.1007/s00264-022-05570-3

**Published:** 2022-09-16

**Authors:** Jobe Shatrov, Benoit Coulin, Cécile Batailler, Elvire Servien, Bill Walter, Sebastien Lustig

**Affiliations:** 1 Orthopaedics Surgery and Sports Medicine Department, FIFA Medical Center of Excellence, Croix-Rousse Hospital, Lyon University Hospital, Lyon, France; 2 Sydney Orthopaedic Research Institute at Landmark Orthopaedics, St. Leonards, Sydney, Australia; 3 University of Sydney, St Leonards, Sydney, Australia; 4 Royal North Shore Hospital, St Leonards, Sydney, Australia

**Keywords:** Total knee arthroplasty, Trochlea, Patellofemoral, Kinematic alignment, Functional alignment, Mechanical alignment

## Abstract

**Purpose:**

The ability of kinematic alignment (KA) to consistently restore trochlea anatomy in total knee arthroplasty (TKA) is unknown despite recreation of constitutional anatomy being its rationale for use. The purpose of this study was to assess if alignment choice in TKA effects the ability to restore the native trochlea groove.

**Methods:**

One hundred and twenty-two consecutive patients undergoing robotic-assisted TKA using the MAKO image-based robotic platform had simulated femoral components placed according to kinematic, mechanical and functional alignment principals. Implant position and trochlea restoration between groups were compared. Restoration was assessed by shift (medial–lateral) and depth relative to the native groove from three consistent points; full extension (0°), mid-flexion (30°–40°) and deep flexion (70°–80°).

**Results:**

Three hundred and sixty-six alignment options were analysed. Femoral alignment was significantly different between groups. Of KA, 13.1% compared to 3.3% of FA plans were outside safe coronal boundaries. The trochlear groove was translated the most by MA compared to KA and FA (full extension, MA 7.84 ± 1.99 mm lateral to the native groove, KA 6.40 ± 2.43 mm and FA 6.88 ± 1.74 mm, *p* ≤ 0.001). In full extension, FA most closely restored the trochlear groove depth in all three positions of flexion.

**Conclusion:**

Alignment philosophy led to significant differences in trochlea groove recreation. A kinematically placed femoral component led to positioning considered unsafe in over 13% of cases. A functionally placed femoral component most closely restored trochlea depth in all three positions of flexion.

## Introduction

As many as 50% of patients experience ongoing symptoms following total knee arthroplasty (TKA) [1]. These findings have led vigorous debate regarding alignment philosophy’s in TKA [2, 3], which have focussed on the tibio-femoral compartment with less emphasis on the patellofemoral joint. Modern arthroplasty data demonstrate more than 15% of patients suffer from clinically significant patellofemoral dysfunction following TKA even when the patella is resurfaced [4]. The behaviour of the patella is largely dictated by the position of the trochlea which in TKA is a product of femoral component design and positioning [5]. Restoring constitutional trochlea anatomy may lead to more physiologic patellofemoral kinematics and improve function of the extensor mechanism following TKA.

In order to improve function and satisfaction in TKA, alignment philosophy’s that restore constitutional alignment have been proposed. Insall et al. first described mechanical alignment (MA) as a technique for TKA [6]. In 2008, kinematic alignment (KA) was described by Howell and aimed to restore the kinematics of the native pre-osteoarthritic knee [7, 8]. More recently, functional alignment (FA) was described [9, 10], which considers the patients soft tissue laxity in flexion and extension and adjusts the implant position within defined boundaries to achieve balanced compartments. This technique utilises robotic tools and also considers the native trochlea groove when adjusting the position of the femoral component.

The ability of KA to consistently restore native trochlea anatomy in TKA is unknown despite recreation of constitutional anatomy being its rationale for use. Previous work concluded that neither MA or KA restored the sulcus orientation, although a KA groove more closely resembled the native state [11]. However, these findings were based on 13 cases and have not been compared to the novel technique of FA. A major concern with KA is that femoral component positioning in some instances may result in extreme positions [3] of femoral valgus or internal rotation and lead to patellofemoral complications.

The purpose of this study was to assess if alignment philosophy effects the ability to restore the native trochlea groove when using a standard femoral implant. The hypothesis was that a kinematically placed femur would best restore the native trochlea groove but result in a significant number of femoral components being placed outside safe limits for rotational alignment.

## Materials and methods

### Ethics

Data collection and analysis were carried out in accordance with MR004 Reference Methodology from the Commission Nationale de l'Informatique et des Libertés (Ref. 2,226,075).

### Study design and participants

This was a prospective matched cohort study of 169 consecutive patients presenting to a single centre that underwent robotically assisted TKA using the MAKO robotic platform and Triathlon Implant (Stryker, Mahwah, USA) between March 2020 and February 2022.

**Fig. 1:**
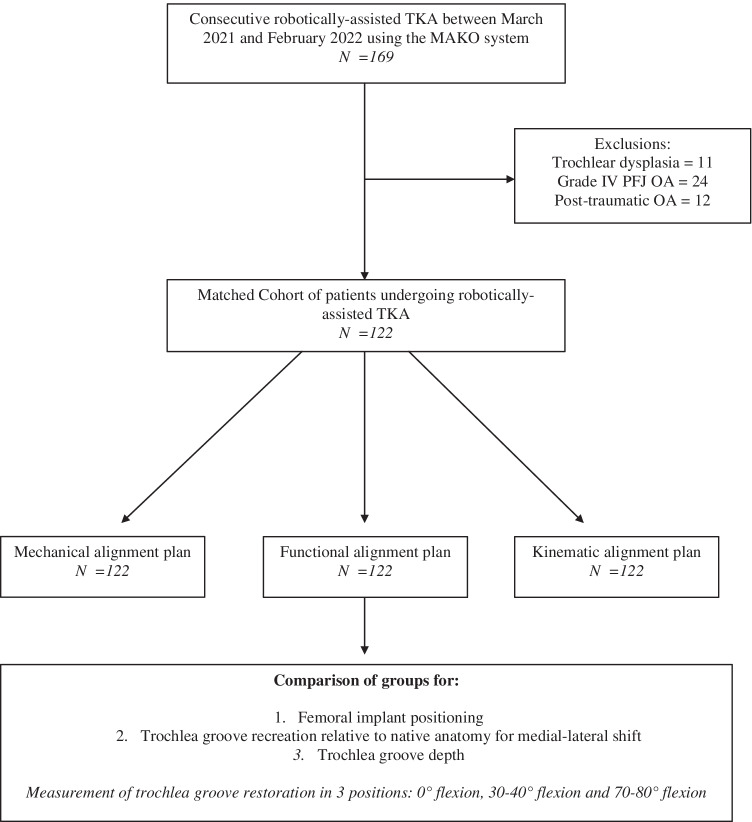
Flowchart of patient selection

Trochlea groove recreation was assessed in patients undergoing robotic-assisted TKA using a FA philosophy. Patients with trochlea dysplasia, intra articular femur fracture, stage 4 patellofemoral arthritis according to the IWANO classification [12] or previous femoral osteotomy were excluded. All surgeries were performed by two surgeons (SL and CB) with more than five years’ experience using robotic assistance for TKA. Both surgeons perform over 200 TKAs annually and have clinical experience with all three alignment philosophies described in the study. Both surgeons were blinded to the outcome of the trochlea recreation using a KA and MA philosophy. A study flow chart is shown in Fig. 1. A final cohort of 122 patients were included in the final analysis.

### Implant sizing

The MAKO system of anatomical landmark registration has been previously described [13]. A 3D model of the implant and bony anatomy is verified and has been previously shown to be within 1 mm of accuracy [14, 15]. The femoral implant was selected using posterior referencing, selecting the size that minimised over-stuffing the patellofemoral joint, strictly avoiding any mediolateral overhang whilst not notching the femur.

### Safe zones for implant position

Implant position outside following thresholds were defined as unsafe and are based on previous literature and guidelines [9, 16]. Limit for femoral flexion was 10° and femur coronal positioning great between 6° valgus and 3° varus. For rotation, limitation relative to the Transepicondylar axis (TEA) was set at 3° external rotation (ER) to 6° IR. A second threshold of 3° IR was also considered as this has previously been associated with patellofemoral failure following TKA [17].

### Implant position and radiographic measurements

#### Radiographic measurement

All measurements were made using PACS digital radiographic software (Centricity ™, GE healthcare, Chicago, USA) and measured by the same evaluator (J.S). The hip-knee-ankle HKA angle, the lateral distal femoral angle (LDFA) and the medial proximal tibial angle (MPTA) were measured pre post-operatively using previously described techniques [18].

#### Trochlea groove measurements

**Fig. 2:**
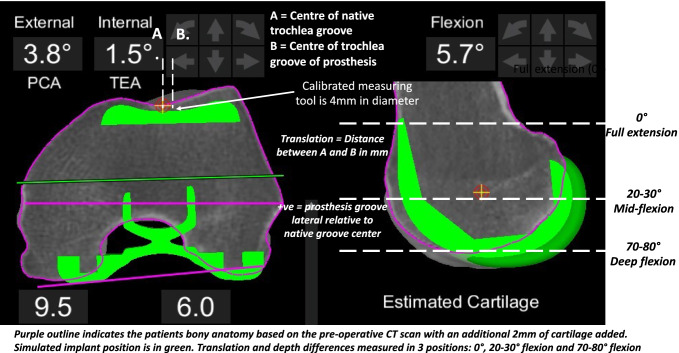
Trochlea groove measurement method—the difference between the trochlea groove centre of the prosthesis position and patients’ native bone was measured for translation and over-stuffing in 3 positions; full extension (0°), mid-flexion (30–304° flexion) and full flexion (70–80°)

The deepest point of trochlea groove on the prosthesis was compared to the deepest point of the native trochlea on pre-operative CT scan using a calibrated tool that allows measurements in 1 mm increments. Restoration was assessed on the MAKO implant planning interface by measuring translation (medial–lateral) on coronal slices and depth on sagittal slices (Fig. 2).

The position of the prosthetic trochlear was compared to the patients native bony trochlear in three locations on the femoral prosthesis. The first location was the earliest axial slice where the prosthetic sulcus was visible (start of the trochlea groove), the second was the point at which the anterior flange met the anterior chamfer on the sagittal profile of the prosthesis and the final position was the last axial slice where the prosthesis groove was visible (most distal part of the trochlea groove). During ten consecutive TKA’s, the flexion range at which the patella engaged each of these three positions was recorded and named according to the flexion range that the patella engaged the prosthesis (Fig. 2); full extension (0°), mid-flexion (30–40°) and deep flexion (70–80°).

Patients were grouped based on alignment philosophy: MA, KA, FA and compared. For the first 100 cases, measurements were recorded twice by each investigator to compare inter-rate reliability and confirm accuracy of measurements.

The intraclass correlation coefficient (ICC) was calculated to measure the reliability (intra and inter observer) of trochlea groove over-/under-stuffing and translation measurements between the two observers in 100 consecutive knees in each group. The intraclass coefficient measurements for trochlea depth and translation were above 0.75 (0.935 and 0.896 respectively), indicating a high degree of intra and inter observer reliability of the two measurements.

#### Planning alignment philosophy groups

**Fig. 3:**
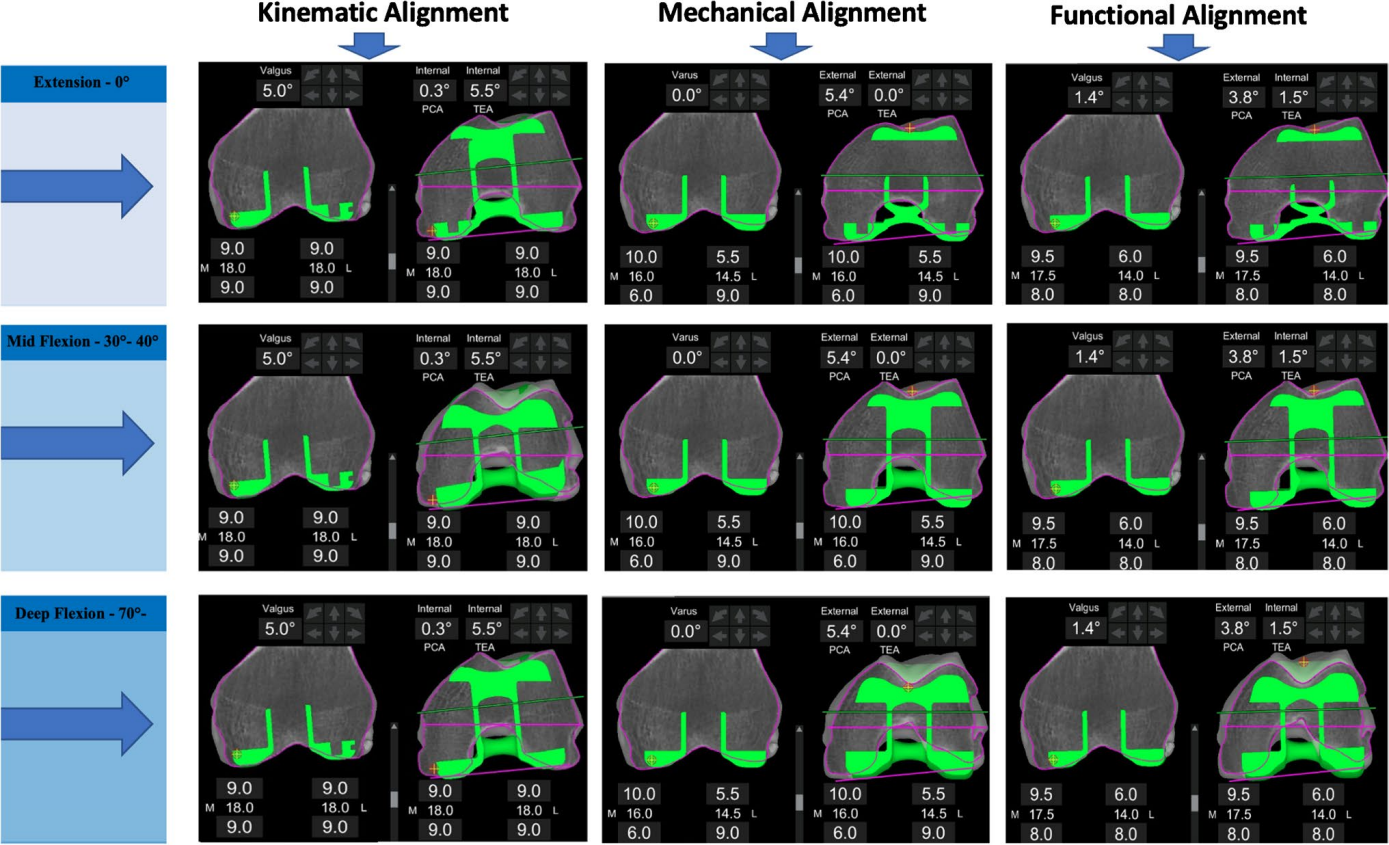
Comparative analysis of trochlea recreation using 3 alignment methods. An example of differences in trochlea recreation using three different alignment techniques for the same patient. Native anatomy outlined by purple line; prosthesis position outlined by green. Translation and over or under-stuffing between the anatomy and prosthesis were calculated from the same position of the prosthesis which corresponded to (0°, 30–40° flexion and 70–80° flexion)

Planning software was used in a mode that adds 2 mm of cartilage thickness to the subchondral bony surface on the CT scan—an assumption previously described in the use of KA [19]. The femoral component was placed according to principles of previously described alignment techniques [20] (Fig. 3).

For the FA, implant positioning is adjusted within limits to achieve an overall alignment and soft tissue laxity goal. Following the assessment of laxity intra-operatively, virtual gaps are assessed to determine if the original plan will deliver a balanced knee. Final adjustments to the femoral component position aim to minimise deviations from the constitutional groove as much as possible. For KA, the distal femur and proximal tibia resections were planned parallel to the native joint line accounting for a 2-mm cartilage thickness, and a similar symmetric posterior condylar (PCA) resection was performed which set femoral rotation parallel to the PCA. Resection depths are based on the thickness of the implant [21]. For MA, the distal femoral cut was planned at 0° to the mechanical axis of the femur and the rotation set at 0° to the TEA (Fig. 3).

### Statistical analysis

Baseline characteristics were described using mean and standard deviation for continuous measures. Data distribution and equality of variances were tested using Shapiro–Wilk and Levine’s test respectively with a significance level set at *P* > 0.05). Normally distributed data was analysed using analysis of variance (ANOVA) and skewed data using Kruskal–Wallis test. Post hoc analysis was performed using Bonferroni’s test. Significance was set at *p* < 0.05 for all tests. statistical analysis was performed using SPSS (IBM, version 18.0).

## Results

### Patient characteristics

Following exclusions, a total of 122 patients were available for a matched comparative analysis. Cohort characteristics are summarised in Table 1.

**Table 1:** Cohort characteristics

	*N*	Mean	SD	Min	Max
*Age*		67.2	7.9	39.0	84.0
*BMI*		29.1	4.9	21.0	49.0
*HKA angle*		175.2	5.6	164	192
*LDFA*		88.2	3.0	79	99
*MPTA*		86.5	3.3	77	95
*Fixed flexion deformity*		-3.4	6.5	-23	16
*Clinical ROM pre-op*		122.5	12.6	90	140
*Iwano Gr*		Frequency	%		
	Normal	5	4.1		
	1	58	47.5		
	2	31	25.4		
	3	28	22.9		
*Albhack Gr*					
	2	2	1.6		
	3	99	81.1		
	4	21	17.2		
*Gender*					
	Female	65	53.2		
	Male	57	46.7		
*ASA grade*					
	1	9	7.4		
	2	92	75.4		
	3	21	17.2		
*Morphotype*					
	Valgus	12	9.8		
	Varus	110	90.2		

*LDFA*, lateral distal femoral angle; *MPTA*, Medial proximal tibial angle; *HKA*, hip-knee-ankle angle; *BMI*, body mass index; *ROM*, range of motion; *Iwano grade*, severity of patellofemoral arthritis on pre-operative imaging

### Femoral component positioning between alignment groups

**Fig. 4:**
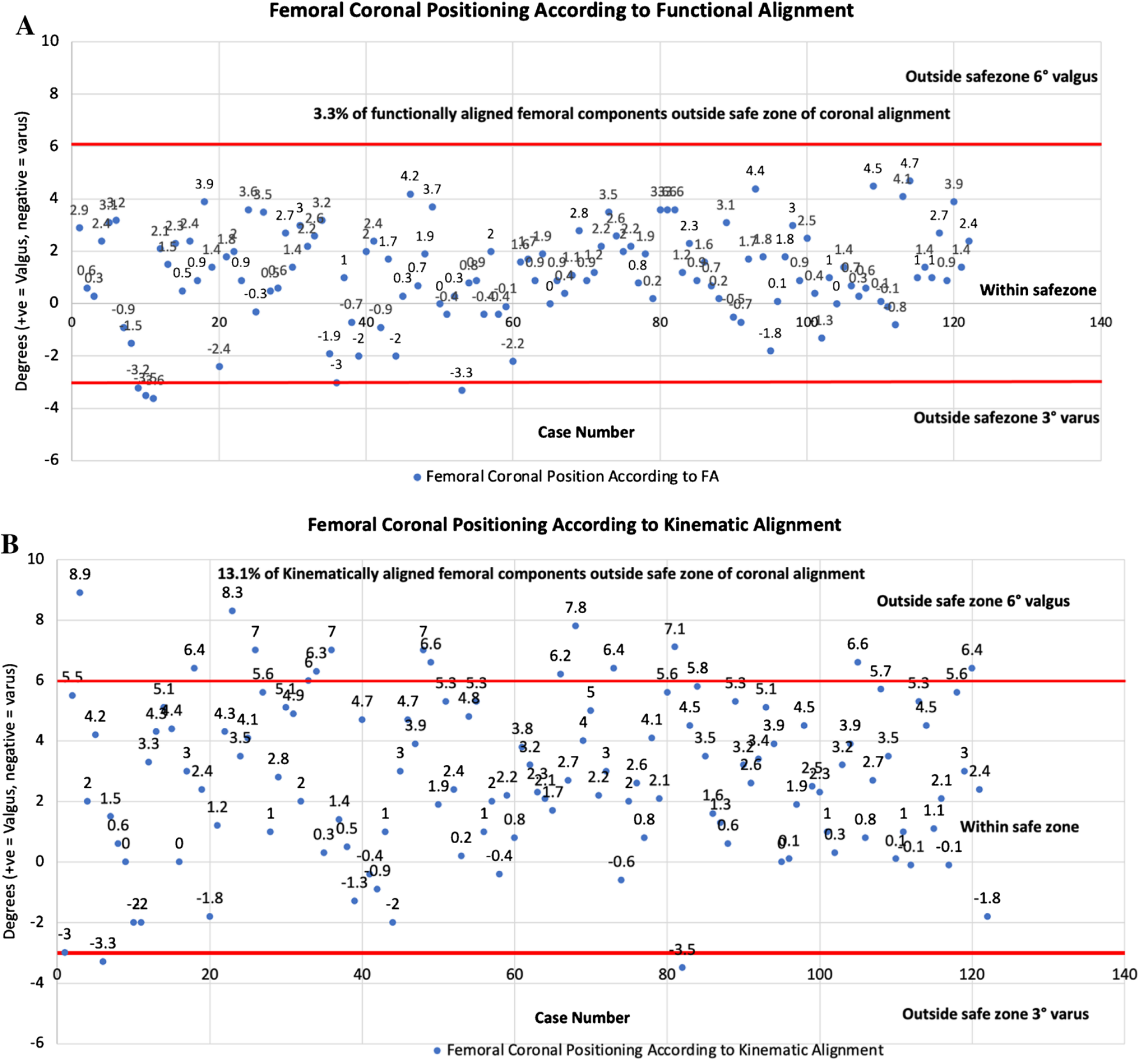
Comparative summary of coronal implant positioning for functional and kinematically aligned femoral component

**Fig. 5:**
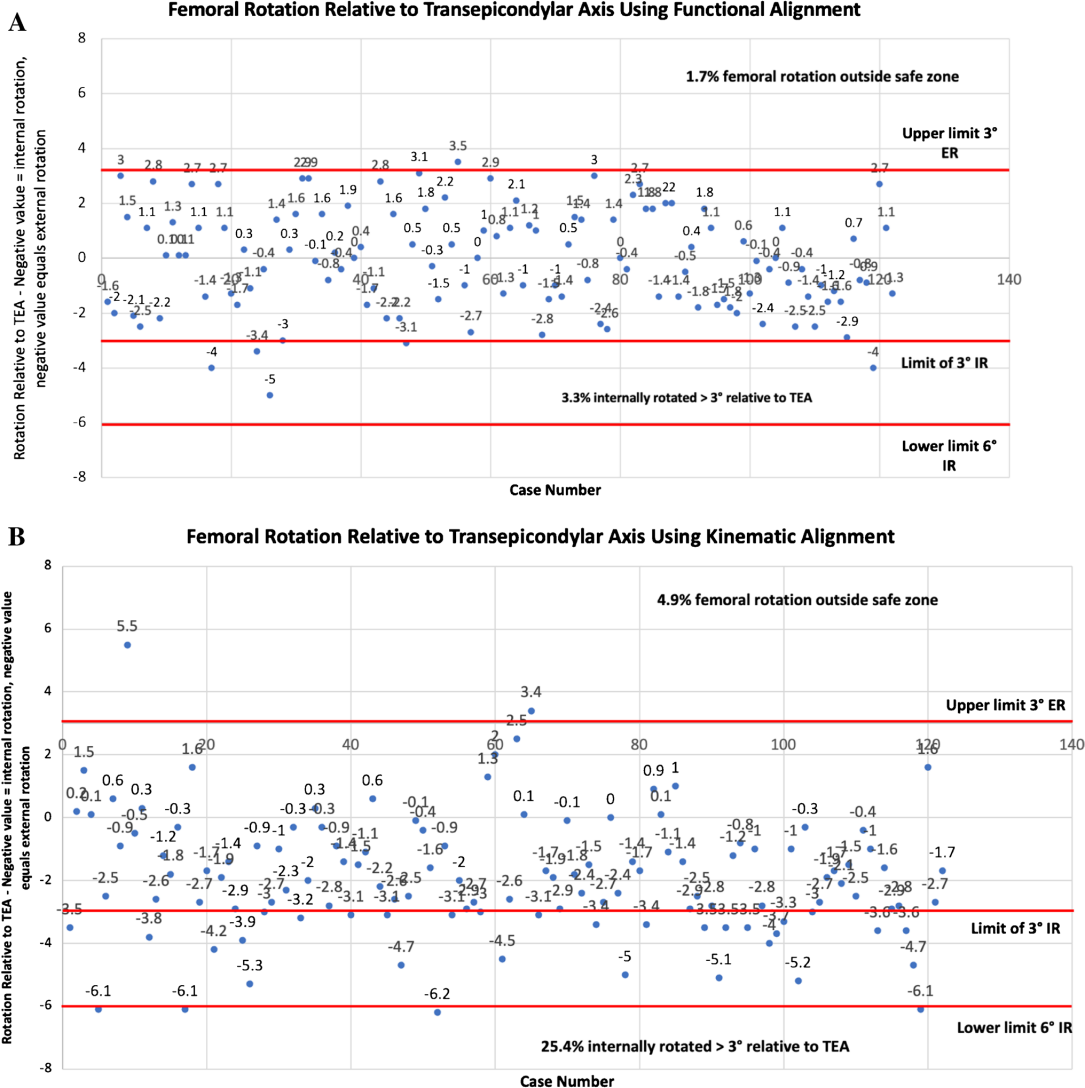
Comparative summary of rotational implant positioning for functional and kinematically aligned femoral components

Femoral component positioning according to alignment philosophy is summarised in Table 2 and group comparisons in Table 3. Of KA, 13.1% and 4 (3.2%) of FA plans for femoral coronal positioning set the implant outside safe zone limitations (Fig. 4A and B)**.** Majority of these occurred by placement of femoral component in more than 6° of valgus. For femoral rotation using the TEA as reference, KA placed 3.3% outside of a safe zone limit of 3° ER to 6° IR, with 25% being placed in more than 3° IR (Fig. 5A), compared to FA which had 1.6% outside the safe zone and 4.1% being placed in more than 3° IR (Fig. 5B).

**Table 2:** Femoral component positioning by alignment philosophy

Implant position	Philosophy	Mean	SD	Min	Max
Femoral coronal position*	KA	3.0	2.6	− 3.5	10.0
	MA	0.0	0.0	0.0	0.0
	FA	1.1	1.8	− 3.6	4.7
*Femur flexion*	KA	6.0	2.4	0.0	10.0
	MA	5.9	2.3	0.0	10.0
	FA	6.5	2.5	0.0	10.0
Femoral PCA**	KA	− 0.2	0.5	− 3.0	2.4
	MA	2.3	2.0	− 3.8	7.8
	FA	2.0	1.6	− 1.0	5.3
Femoral TEA**	KA	− 2.1	2.0	− 7.2	5.5
	MA	0.0	0.0	0.0	0.0
	FA	− 0.1	1.9	− 5.0	3.5

^*^Indicates relative to the mechanical axis of: + ve = valgus, − ve = varus

^**^Rotation relative to the named landmark: − ve = internal rotation; + ve = external rotation

All measurements in degrees

**Table 3:** Comparison of femoral component positioning by alignment philosophy

Dependent variable	Alignment group comparison	Mean difference**	Adjusted *P*-value*
*Femoral coronal position*^*a*^	KA vs. MA	3.0	** > *0.001***
	KA vs. FA	2.2	** > *0.001***
	FA vs. MA	0.8*	***0.005***
*Femur flexion*	KA vs. MA	0.1	*1.000*
	KA vs. FA	− 0.5	*0.424*
	FA vs. MA	0.5	*0.424*
*Femoral rotation, PCA*^*b*^	KA vs. MA	− 2.9*	** > *0.001***
	KA vs. FA	− 2.2*	** > *0.001***
	FA vs. MA	2.2*	** > *0.001***
*Femoral rotation, TEA*^*b*^	KA vs. MA	− 2.1*	** > *0.001***
	KA vs. FA	− 2.0*	** > *0.001***
	FA vs. MA	2.0*	** > *0.001***

^*^The mean difference is significant at the 0.05 level. Post hoc analysis using Bonferroni’s multiple comparison

^**^Differences in degrees

^a^Relative to the mechanical axis of: + ve = valgus, − ve = varus

^b^Rotation relative to the named landmark: − ve = internal rotation; + ve = external rotation

### Trochlear groove translation between alignment groups

**Fig. 6:**
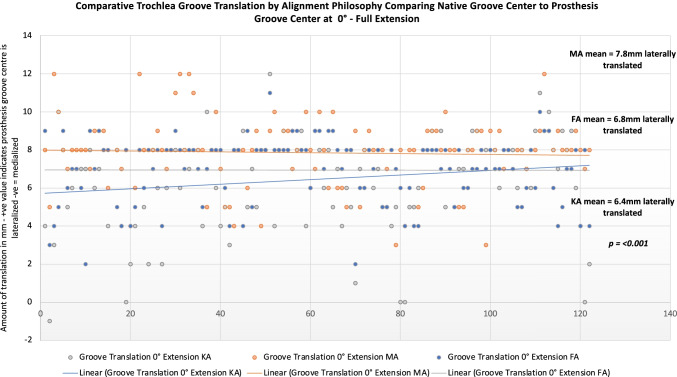
Comparative trochlea groove translation by alignment philosophy. In full extension, all alignment philosophies led to a trochlea groove that was lateral to the native state, with mechanical alignment creating the largest deviation from the constitutional anatomy

Trochlear groove translation between alignment philosophy’s is summarised in Table 3 and comparison of trochlear groove translation for alignment philosophy’s is summarised in Tables 4 and 5. All alignment philosophy’s resulted in a trochlear groove position that started lateral relative to the patients native trochlea groove. A MA femoral component deviated the furthest (more lateral) from the patient’s native groove. The mean trochlear groove translation was significantly different in full extension between KA and MA, mean difference − 1.4 mm (more lateral), *p* =  < 0.001, but not KA and FA mean − 0.48 (n.s). Groove translation difference between FA and MA in full extension was also significantly different, mean difference − 0.96 mm *p* = 0.002. In mid-flexion, there was a significant difference in groove translation between KA and MA mean − 0.75 mm, *p* = 0.003, but not between KA and MA*.* Case-by-case comparison and distribution of groove translation between alignment philosophy’s is shown in Fig. 6.

**Table 4:** Trochlear groove translation comparisons between alignment philosophy’s

Position**	Alignment	Mean*	SD	Min	Max	P*
*Full extension, 0°*	KA	6.4	2.4	− 1.0	12.0	
	MA	7.8	1.9	− 4.0	12.0	
	FA	6.8	1.7	2.0	11.0	***p* ≥ *0.001***
*Mid-flexion, 20–30°*	KA	− 0.4	1.9	− 6.0	5.0	
	MA	0.2	1.7	− 8.0	5.0	
	FA	− 0.2	2.2	− 5.0	5.0	***p* = *0.003***
*Deep flexion, 70–80°*	KA	− 0.8	1.2	− 4.0	3.0	
	MA	− 0.7	1.3	− 5.0	3.0	
	FA	− 1.04	1.7	− 5.0	3.0	***p* = *0.396***

^*^Kruskal–Wallis test, post hoc multiple comparisons performed using Bonferroni’s test difference is significant at the 0.05 level

^*^Amount of translation of the centre of the prosthesis groove relative to the patients native groove: positive values indicate the prosthesis groove is *lateral*, and negative values indicates the prosthesis groove is *medial* to the native groove centre

^**^The difference between the prosthesis groove and native groove centre was assessed in three positions of the trochlea: full extension, mid-flexion and deep flexion

**Table 5:** Trochlear groove translation comparisons between alignment philosophy’s

Position	Alignment group comparison	Mean difference**	P*
*Full extension, 0°*	KA vs. MA	− 1.4*	** > *0.001***
	KA vs. FA	− 0.4	*0.248*
	FA vs. MA	− 0.9*	***0.002***
*Mid-flexion, 30–40°*	KA vs. MA	− 0.7*	***0.003***
	KA vs. FA	− 0.2	*1*
	FA vs. MA	− 0.5	*0.095*
*Deep flexion, 70–80°*	KA vs. MA	− 0.1	*1*
	KA vs. FA	0.2	*1*
	FA vs. MA	− 0.3	*0.527*

^*^Kruskal–Wallis test, post hoc multiple comparisons performed using Bonferroni’s test difference is significant at the 0.05 level

^**^Amount of groove centre translation between the prosthesis and native bone: + ve values indicate prosthesis groove is *lateral* to the native groove, − ve values indicate prosthesis groove is *medial* to the native groove. All measurements in mm’s

### Trochlear groove depth between alignment groups

**Fig. 7:**
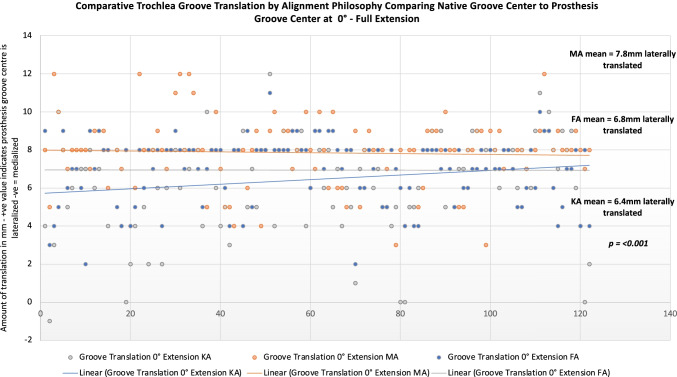
Comparative trochlea groove depth restoration by alignment philosophy. In full extension, all alignment philosophies led to a trochlea groove that was slightly over-stuffed compared to the native state, with functional alignment most closely resembling the constitutional groove depth

A comparison of trochlear groove depth comparisons between alignment philosophy’s is summarised in Table 6 and comparisons in Table 7. In full extension (0°), all alignment philosophy’s resulted in an implant position that was over-stuffed compared to the native groove. KA over-stuffed the most, 1.36 mm ± 1.16 mm and FA the least, 0.83 mm ± 1.18 mm (*p* = 0.002). In flexion, all philosophies had a mean under-stuffing compared to the native groove. Under-stuffing was greatest with KA followed by MA and FA (*p* <  = 0.001). Case-by-case comparison and distribution of groove depth between alignment philosophy’s is shown in Fig. 7.

**Table 6:** Trochlear groove depth comparisons between alignment philosophy’s

Position	Alignment	Mean**	SD	Min	Max	P*
*Full extension, 0°*	KA	1.3	1.1	− 2.0	5.0	** > *0.001***
	MA	1.2	1.2	− 4.0	5.0	
	FA	0.8	1.1	− 2.0	6.0	
*Mid-flexion, 20–30°*	KA	− 2.1	1.6	− 7.0	3.0	***0.041***
	MA	− 1.6	1.7	− 6.0	4.0	
	FA	− 1.7	1.9	− 6.0	4.0	
*Deep flexion, 70–80°*	KA	− 2.3	2.4	− 9.0	6.0	** > *0.001***
	MA	− 1.1	2.6	− 9.0	5.0	
	FA	− 0.1	2.5	− 6.0	5.0	

^*^One-way ANOVA analysis. Significance at the 0.05 level

^**^Difference between prosthesis position and patient’s native groove position measured in mm’s: + ve values indicate implant is proud, or “over stuffed,” − ve value indicate the implant is below the patients native anatomy or “under stuffed”

**Table 7:** Trochlear groove depth comparisons between alignment philosophy’s

Dependent variable	Alignment group	Mean difference**	P*
*Trochlea depth–full extension, 0°*	KA vs. MA	0.1	*1.000*
	KA vs. FA	0.5	***0.002***
	FA vs. MA	− 0.5	***0.010***
*Trochlea depth mid-flexion, 20–30°*	KA vs. MA	− 0.5	*0.060*
	KA vs. FA	− 0.4	*0.164*
	FA vs. MA	− 0.1	*1.000*
*Trochlea depth, 70–80°*	KA vs. MA	− 1.2	** > *0.001***
	KA vs. FA	− 2.2	** > *0.001***
	FA vs. MA	1.0	***0.007***

^*^Multiple pair-wise comparison with Bonferroni test. Mean difference is significant at the 0.05 level

^**^Difference between prosthesis position and patient’s native groove position measured in mm’s: + ve values indicate implant is proud, or “over stuffed,” − ve value indicate the implant is below the patients native anatomy or “under stuffed”

## Discussion

Choice of alignment philosophy led to significant variations in trochlear groove restoration. MA resulted in the greatest translation shift and KA created the most under-stuffing overall. FA and KA were equally consistent in reproducing the trochlear groove in terms of translation; however, KA resulted in femoral coronal component positioning that is considered unsafe in 13.2% of cases, compared to FA in 3.7% of cases. A KA philosophy also resulted in a femoral component that was IR beyond 3° relative to the TEA in more than 25% of cases compared to 3.3% with a FA philosophy. These findings confirm our hypothesis that a KA placed femoral component restores the constitutional trochlea groove alignment but frequently results in unsafe coronal or axial implant positioning. These findings may explain why patellofemoral complications are the most common reason for revision in KA TKA [22–24].

### Coronal positioning

In the current study, more than 13% of patients in the KA group had a femur positioned in excess of 6° valgus. This is the upper limit of valgus considered safe using the prosthesis used in this study. Importantly, only 3% of FA aligned femurs were outside the safe range, none of which were in valgus. Aligning the femoral component according to the LDFA results in kinematically aligned femur increasing when the valgus obliquity of the distal femur increases [11]. This reduces the efficiency of the valgus angle built into the prosthesis design (such as 7°) that is designed to facilitate a soft capture of the patella in early flexion [25]. This effect will at a certain threshold become clinically relevant and may explain why patellofemoral problems in KA TKA have been more commonly observed in patients with valgus phenotypes [22]. Such a problem could be dealt with either through increasing the valgus angle in the prosthesis design, thus creating a wider capture in the trochlea groove, or by staying within defined safe zones such as that of FA. Importantly, this study demonstrated that FA remained within the coronal safe zone of 3° varus to 6° valgus in 97% of cases, and did not result in compromise of the trochlea groove compared to KA.

### Axial positioning

The most common cause of revision following KA TKA are patellofemoral problems (instability and pain) [22–24]. Femoral malrotation is a significant contributor to patellofemoral instability. Berger et al. examined patients with patellofemoral failure following TKA and reported more than 50% had a femoral component > / + 3° IR relative to the TEA. The results of the current study show extreme positions of femoral component IR occur frequently when systematically following the PCA for femoral rotation, with KA resulting in the femoral component being IR beyond 3° in more than 20% of cases. This observation may also be partially responsible for the patellofemoral problems encountered with KA. However, unlike the aforementioned coronal issue which may be addressed through changing of implant design, the wide variation seen in the rotational axis of knees is more complex to deal with and an individualized approach such as FA where limitations to rotation are observed seems sensible. Importantly, FA which also aims to restore constitutional alignment but uses defined boundaries was not inferior to KA in terms of groover restoration and in this series had only 3.2% of patients outside of coronal and 1.7% outside of rotational safe zones.

### Translation of the trochlea

Little data is published regarding the effect of alignment philosophy on groove translation in TKA. Previously, Rivière et al. examined the effect of KA in 10 arthritic knees, demonstrating the prosthetic groove was 2 mm lateral in extension, neutral with the native groove at 40° flexion and ended medial at 90° flexion [26]. In the current study, use of a MA technique resulted in a groove that was 7.8 mm lateral, which was significantly different to FA (6.8 mm) and KA (6.4 mm). The reason for the higher amount of groove translation in extension may be due to differences in prosthesis design, study sample size, and also differences in medial–lateral positioning of the prosthesis. In the previous study, the prosthesis was centred on the intercondylar notch, whereas in the current study a maximum lateral position without any overhang was adopted. Similar to the previous study, we also observed that in mid-flexion (30–40° flexion) the prosthetic groove tended to be co-aligned with the native groove, finishing in a medialised position in deeper flexion. This reflects the complex geometry of the trochlea groove, being slightly C-shaped in the coronal plane [27]. These findings also show the effect of alignment philosophy on trochlea groove translation are greatest in full extension and decrease through flexion, and that KA produces a trochlea groove that lies closest to the native groove compared to MA. Whilst deviations in the groove are undesirable due to the alteration of retinacular tension, clinically relevant thresholds are yet to be defined and should be the focus of future research.

### Over and under-stuffing the trochlea

Data examining the effect of alignment philosophy on over- and under-stuffing of the trochlea is also limited. Previously, a study of ten arthritic knees demonstrated that KA led to a mean under-stuffing of 4 mm in extension, 4–5 mm under-stuffing in mid-flexion but no under-stuffing in 100° flexion. In the current study, all alignment philosophy’s had a tendency to slightly overstuff the trochlear in full extension whilst under-stuffing through mid-flexion flexion. In deep flexion, FA resulted in a component flush with the native groove, whilst MA and KA resulted in statistically more under-stuffing (1.0 mm and 2.2 mm respectively). These findings demonstrate that trochlea depth restoration is difficult with the use of a standard design implant. Differences larger than 2 mm have been used to define over-stuffing or under-stuffing [28, 29]; however, future research should examine the clinical effect of under-stuffing the trochlea in mid-flexion, which in biomechanical studies has been shown to result in a reduction in the quadriceps lever arm [30].

### Limitations

This study has weakness’s. Firstly, all comparisons were simulated and performed in silico. However, the purposes of this study were to examine the recreation of the native trochlea groove which can be done using the CT-based planning to within 1 mm and 1° accuracy [14, 15], meaning the measurements observed in this study are an accurate reflection of the in vivo conditions. Furthermore, all femoral components were placed in a manner to best replicate the native groove visible on the CT scan; therefore, all data depicts the best possible scenario for each technique and may even underestimate the differences between groups. Furthermore, whilst differences in ability to restore the sulcus could be demonstrated, the effect on clinical outcomes remains unknown and will need to be the focus of future research. Finally, this study utilised previously described safe zones on implant limits [9], and these limits are subject to ongoing debate in TKA and may vary over time and between implant designs.

## Conclusion

Alignment choice has a significant effect on the ability to restore the constitutional trochlea in TKA when using a standard femoral component. MA translates the groove furthest from the native anatomy. KA and FA have a similar ability to restore the constitutional trochlear groove; however, KA requires unsafe coronal implant positioning in at least 13% of cases and an internally rotated femoral component beyond 3° in more than 25% of cases. These findings have implications for the function of the patellofemoral joint, implant design, and choice of alignment philosophy in TKA.

## Data Availability

All data related to this study is available upon request.
